# Incorporation of Cobalt‐Cyclen Complexes into Templated Nanogels Results in Enhanced Activity

**DOI:** 10.1002/chem.201503946

**Published:** 2015-12-10

**Authors:** Ana Rita Jorge, Mariya Chernobryva, Stephen E. J. Rigby, Michael Watkinson, Marina Resmini

**Affiliations:** ^1^ Department of Chemistry and BiochemistrySchool of Biological and Chemical SciencesQueen Mary University of LondonMile End RoadLondonE1 4NSUK; ^2^ Faculty of Life SciencesManchester Institute of Biotechnology131 Princess StreetManchesterM1 7DNUK

**Keywords:** cyclen, enzyme mimics, molecular imprinting, nanogels, phosphatase

## Abstract

Recent advances in nanomaterials have identified nanogels as an excellent matrix for novel biomimetic catalysts using the molecular imprinting approach. Polymerisable Co‐cyclen complexes with phosphonate and carbonate templates have been prepared, fully characterised and used to obtain nanogels that show high activity and turnover with low catalytic load, compared to the free complex, in the hydrolysis of 4‐nitrophenyl phosphate, a nerve agent simulant. This work demonstrates that the chemical structure of the template has an impact on the coordination geometry and oxidation state of the metal centre in the polymerisable complex resulting in very significant changes in the catalytic properties of the polymeric matrix. Both pseudo‐octahedral cobalt(III) and trigonal‐bipyramidal cobalt(II) structures have been used for the synthesis of imprinted nanogels, and the catalytic data demonstrate that: i) the imprinted nanogels can be used in 15 % load and show turnover; ii) the structural differences in the polymeric matrices resulting from the imprinting approach with different templates are responsible for the molecular recognition capabilities and the catalytic activity. Nanogel **P1**, imprinted with the carbonate template, shows >50 % higher catalytic activity than **P2** imprinted with the phosphonate.

## Introduction

Catalysis of energetically demanding reactions, such as phosphate ester hydrolysis, has challenged researchers for many years.^1^, ^2^ Whilst Nature has evolved enzymes that efficiently promote such reactions, their applications are often limited.^3^ Among the different approaches investigated for the development of synthetic alternatives, metal complexes have attracted considerable interest and have resulted in a number of impressive achievements.^4^ For example, lanthanide‐based systems containing Ce^IV^ are some of the most active inorganic catalysts reported,^5^ but their complexes are often insoluble at environmentally relevant pH and their enhanced reactivity presents a risk of indiscriminate damage to cellular components in vivo.^6^ In parallel, small molecule complexes of transition metals continue to be widely studied.^7^ Although the rational design of simple metal complexes is a key element in the optimisation of their catalytic activity, it has been demonstrated that placing these complexes in low polarity environments that closely mimic the dielectric constant of the active sites of enzymes is very important. Brown et al. showed that triazamacrocyclic bimetallic Zn^II^ complexes can accelerate the cleavage of certain phosphate substrates by 12 orders of magnitude in the presence of methanol,^8^ with accelerations approaching those of natural enzymes.^2^, ^8^ More recently the incorporation of such small molecule catalysts into supramolecular architectures has been shown to significantly enhance their catalytic properties.^9^, ^10^ Of particular interest to us was the report by Scrimin et al., demonstrating that self‐organisation of Zn^II^ complexes of azamacrocyclic ligands on gold nanoparticles resulted in remarkable increases in cleavage efficiency when compared to the free complexes in aqueous solution, an effect attributed to the decrease in polarity of the reaction site.4a

We have successfully demonstrated that colloidal microgels and nanogels can be tuned to achieve high catalytic activity using the molecular imprinting approach, and are able to catalyse chemical reactions, such as carbonate hydrolysis,^11^ aldol condensations^12^ and Kemp eliminations^13^ with good efficiency and selectivity. Given our additional expertise in the synthesis of functionalised macrocyclic amine systems^14^ and the use of their metal complexes as biomimetic catalysts^15^ as well as sensors,^16^ the incorporation of these systems into novel nanomaterials via the imprinting approach appeared to have considerable potential.

The use of metal‐templated sites for molecular recognition and sensing has been previously investigated.^17^, ^18^, ^19^ An essential feature for successful imprinting in polymeric systems is the stability and integrity of the template–monomer complex.^20^ In the case of metal complexes, ligand exchange and fluxionality of metal centres can lead to dynamic changes to the coordination environment of the metal. In‐depth studies of the coordination geometry of the templated complex prior to polymerisation are seldom undertaken,^21^ with a few exceptions,^22^ and it is normally assumed that the complex expected to form, based on the coordination chemistry observed in small molecule analogues, will also be maintained in the polymer‐based macromolecular structure.^20^

Here we report the synthesis of polymerisable Co‐cyclen systems that have been complexed with strategically chosen templates to obtain imprinted nanogels, where the three‐dimensional polymeric matrix around the metal atom mimics the secondary coordination sphere of natural metalloenzymes. The catalytic activity of the different polymers in the hydrolysis of 4‐nitrophenyl phosphate **1** is evaluated, in comparison with the free complexes, and analysis of the results used to highlight the impact of the polymeric matrix on the catalytic activity.

## Results and Discussion

**Figure 1:**
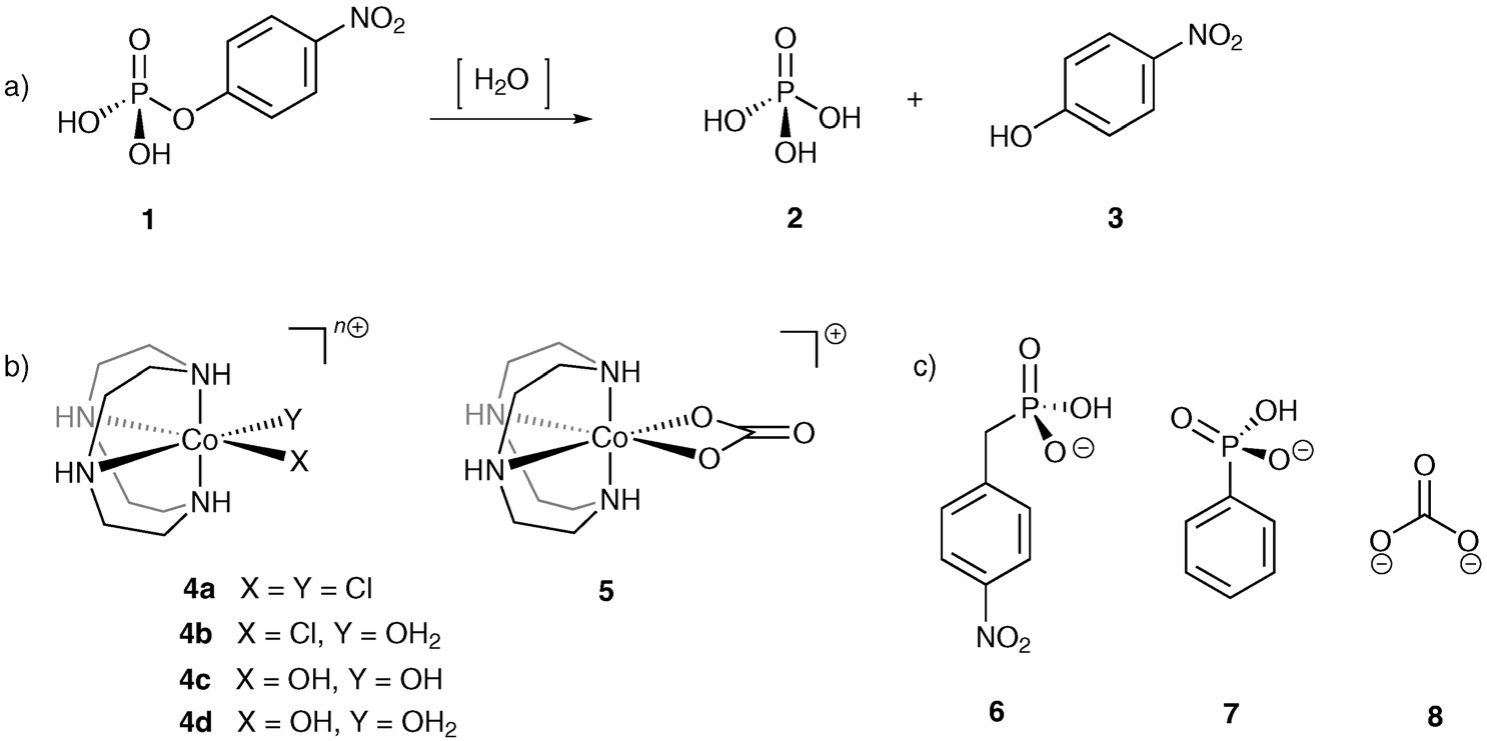
a) Hydrolysis of model substrate **1**; b) structures of model complexes **4** and **5**; c) templates used for the imprinting approach: phosphonates **6** and **7** and carbonate **8**.

We decided to focus our initial investigation on the hydrolysis of 4‐nitrophenyl phosphate **1** (NPP), a model substrate for nerve agents, shown in Figure 1 a, that when hydrolysed releases **2** and the highly chromophoric unit 4‐nitrophenol (**3**), which can be easily monitored by UV/Vis spectroscopy. The choice of catalyst was dictated by our interests in complexes of azamacrocycles,^15^, ^16^ in particular cobalt(III) complexes of the type *cis*‐[Co(cyclen)(X)_2_]^*n*+^,^4^, ^23^ and their ease of synthesis from precursor **5**. Such complexes have been extensively used as ATPase and phosphatase models and remain amongst the most active mimetic systems for phosphate hydrolysis. Chin et al.^24^ first established **4 d** as an active synthetic phosphatase hydrolysis catalyst and reported hydrolysis rate enhancement factors of up to 10^7^, while supercoiled DNA was hydrolysed in the presence of a functionalised derivative of **4**.^25^ However, in all cases turnover was only achieved in the presence of a second metal unit, to release the product.^26^

Several features make these systems attractive for the development of artificial phosphatases. The size of the metal ion and the cyclen ligand cavity results in a rigid and non‐fluxional structure that forces only *cis*‐coordination sites to be present at the cobalt(III) centre.^24^ In contrast, analogous *trans*‐complexes of the closely related cyclam ligand are inactive.^27^ Furthermore, although such *cis*‐complexes have been reported to be active in the hydrolysis of phosphate esters, cobalt(III) complexes are generally considered to be substitutionally inert and should provide a stable interaction with any target template, as well as a very well‐defined primary coordination sphere. We viewed this aspect as a key feature in our imprinting strategy to prevent unwanted ligand exchange and fluxionality of the metal centres which could lead to dynamic changes to the coordination environment of the metal. Additionally, after polymerisation, the template can be readily removed from the cobalt centre under experimental conditions that are well documented.^23^

The choice of template in molecular imprinting plays a key role in influencing the recognition and catalytic properties of the polymers. Traditionally, transition‐state analogues (TSA) have been shown to be good templates, however this is dependent on the catalytic mechanism being mimicked. In this work three compounds were chosen as suitable templates. The first, 4‐nitrobenzyl phosphonate **6** is the cognate substrate analogue to **1** but is not a substrate for the metal complex and provides almost identical chemical features, particularly the *p*‐nitro‐substituent, previously shown to play a key role in molecular recognition.^28^ The other two structurally distinct templates were selected to evaluate the impact of the chemical structure on the molecular recognition properties of the polymer matrix. Phenyl phosphonate **7** has a similar structure to substrate **1** but is lacking the *p*‐nitro‐substituent; it is electronically richer at the oxygen donors and is sterically less demanding. The carbonate template is significantly different, although still a good mimic in terms of its coordination geometry, as its coordination mode in cobalt(III) cyclen complexes is established to be *cis*‐bidentate,^23^ resulting in a pseudo‐octahedral geometry.

**Figure 2:**
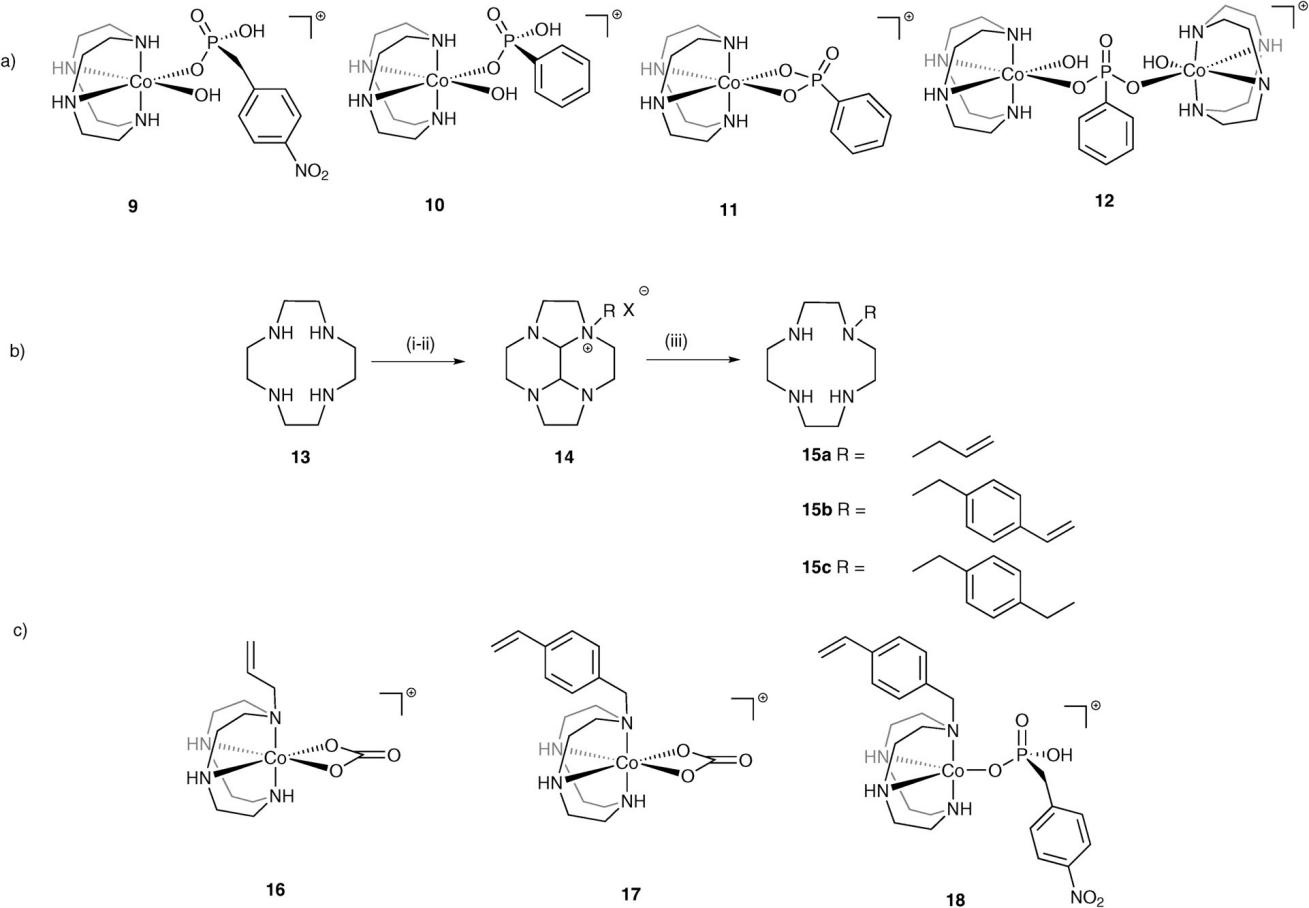
a) Structure of complexes **9**–**12**; b) synthesis of polymerisable and non‐polymerisable ligands **15**; i) glyoxal (40 % aq.), MeOH, 72 %;^[29]^ ii) allyl bromide,^30^ 1‐(chloromethyl)‐4‐vinylbenzene or *p*‐ethyl benzyl bromide^29^, ^31^, ^32^ toluene 93 % and 94 % and acetonitrile 91 %; iii) NH_2_OH (50 % aq.), EtOH, **15 a** 73 %, **15 b** 96 %, **15 c** 18 %;^29^, ^33^ c) co‐polymerisable complexes with carbonate templates **16** and **17** and cognate phosphonate template **18**.

Non‐polymerisable complexes of [Co(cyclen)(L)]^*n*+^ were prepared, where L=(Cl)_2_ (**4 a**), CO_3_^2−^ (**5**), 4‐NPPA (**9**), and PPA (**10**) (Figure 2 a). For **4 a** and **5** the coordination complexes were readily obtained using reported methods,^23^ whilst **9** and **10** were prepared by addition of cobalt(II) chloride and the phosphonate to a solution of cyclen under nitrogen, and the resulting mixture subsequently allowed to oxidize in air.

To better understand the behaviour and stability of the complexes in solution, template **7** was added to **4 a** and the nature, and extent of binding in **10** measured in a range of different solvents, at different temperatures and pH, using ^31^P NMR spectroscopy. Depending on the conditions used, coordination of one, as in **10**, or chelation of both oxygen atoms, as in **11**, to the metal could be favoured; dimerization of cobalt(III) centres also occurs as in **12**. It was found that in water at pD 9, when the complex is in its dihydroxo form [Co(cyclen)(OH)_2_]^+^ namely **4 c** and **7** is a di‐anion, a mixture of monodentate phosphonate **10** (59 %, *K*_a_=1.8×10^−2^ m ^−1^) and chelated phosphonate **11** (39 %) can be found in solution, with a small amount of dimerisation occurring to give **12** (2 %). At 70 °C, the temperature at which polymerisation occurs, this equilibrium is entirely shifted towards **11** (100 %) (see Figure S1 in the Supporting Information). Importantly, addition of HCl results in complete substitution of **7** in the coordination sphere, indicating that this template can be readily removed at room temperature. Further characterisation of **4 a**, **5**, **9** and **10** by UV/Vis, IR spectroscopy, and high‐resolution mass spectrometry supported the identity of all of the complexes and all data were consistent with the same pseudo‐octahedral geometry around the cobalt centre as observed in **5**. UV/Vis spectra gave d–d bands with larger than normal extinction coefficients for octahedral Co^III^ complexes, as previously reported for complexes of this type.^34^

For the synthesis of the polymerisable derivative of **4 d** both vinyl and styrene groups were evaluated. Aoki and co‐workers previously reported the synthesis of **15 b**,^35^ but instead we chose to introduce the alkene bonds via alkylation of the tetracyclic bis‐aminal intermediate by modification of a reported procedure,^29^, ^36^ (Figure 2 b). The first polymerisable complex to be synthesised and characterised was **16**. The coordination geometry appeared to be consistent with the desired pseudo‐octahedral complexes that had been found for the non‐polymerisable analogue with the UV/Vis spectra displaying a single d–d band with a *λ*_max_ at 535 nm. This complex led to unsatisfactory yields in the polymerisation and it was replaced with the styrene unit, that although bringing additional steric bulk to the structure, is known to be more active in radical polymerisation.^37^, ^38^ Complex **17** was prepared and UV/Vis analysis was also consistent with the geometry observed in **5** (see Figure S2).

Coordination of **6** and **7** to the cobalt–styrene monomer was successfully achieved using the same procedure adopted for the preparation of **9** and **10**, although analysis by UV/Vis and ^31^P NMR spectroscopies displayed some anomalies. For **7** the UV/Vis spectrum showed a band at significantly higher wavenumber (580 nm) and a meaningful ^31^P NMR spectrum could not be recorded. Nonetheless high‐resolution mass spectra and IR spectra appeared to be consistent with the formation of the expected molecular species [Co(**15 b**)(**7**)(OH)]^+^. The complex with **6** presented the same effect in the NMR studies but exhibited even more pronounced differences in its UV/Vis spectrum, with the presence of new d–d bands in the region 600–650 nm (Figure S3). This behaviour appeared to be consistent with the presence of a paramagnetic cobalt(II) species. Previous work by Sarther and Blinn,^39^ reported a tetra‐benzylated derivative of cyclen that was coordinated to cobalt(II) resulted in a complex that was resistant to oxidation by electrochemical means, direct aeration or by strong oxidants such as H_2_O_2_.

**Figure 3:**
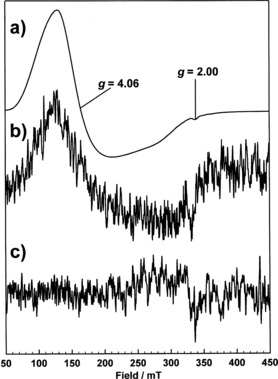
EPR spectra of frozen DMSO solutions of: a) **18**, b) a solution of nanogel **P2** prior to template removal, c) nanogel solution **P2** after template removal at 18 K.

The complex was shown to have the rare five‐coordinate trigonal‐bipyramidal geometry and displayed the same characteristic UV/Vis spectra observed for **18** suggesting that this complex was also five‐coordinate cobalt(II) (Figure 2c). To further substantiate this hypothesis, magnetic moments were measured using the Evans’ NMR method.^40^ This showed that both complexes of ligand **15 b** with templates **6** and **7** contained significant quantities of paramagnetic species in solution, the latter, **18**, apparently being almost exclusively high‐spin cobalt(II) based on its magnetic moment (μ_eff_=4.0 μ_B_). Analysis of both complexes by X‐band CW‐EPR spectroscopy clearly showed them to contain high‐spin cobalt(II). Spectra recorded at 18 K, using 0.5 mW of microwave power and 0.5 mT field modulation are broad and axial with *g* values of approximately 4 and 2 (see Figures 3 a–c and Figure S4 in the Supporting Information)). This is typical of frozen solution spectra arising from the *S*=3/2 d^7^ spin system of the high‐spin cobalt(II) ion having a zero‐field splitting (D) greater than *hυ* at the X band (υ=9.38 GHz).^41^

The trigonal‐bipyramidal geometry is very rare for cyclen compounds, and the use of such strong field ligands normally results in the easy oxidation of cobalt(II) to cobalt(III), as seen for the non‐polymerisable ligands. However, the common octahedral *cis*‐cyclen cobalt(III) geometry already contains strained metal to donor bonds which are presumably further strained through the incorporation of the *N*‐benzyl pendant arm and the phosphonate, resulting in the ligand now behaving as an unusually weak polyamine ligand.^40^ The data suggest that subtle changes in ligand and phosphonate structure result in significant changes to the coordination geometry and oxidation state of the metal centre. Importantly, under the polymerisation conditions (DMSO, 70 °C) the characteristic UV/Vis bands for the five‐coordinate cobalt(II) centre in **18** are unaltered, indicating that this coordination geometry is maintained (see Figure S5). Given that the ligand complex with **7** appeared as a mixture of species, it was not used for the preparation of the nanogels.

Nanogels were synthesised by high‐dilution radical polymerization of complexes **17** and **18**, following our previously reported protocol,^11^ using 80 % ethylene bis‐acrylamide (EBA) as the crosslinker, 10 % acrylamide and 10 % of the polymerisable catalyst and DMSO as the solvent. Template molecules **6** and **8** were then removed from the matrix by washing with dilute HCl. Further dialysis in water resulted in the generation of the catalytically active Co^III^ aqua‐hydroxo species (Figure S6). Two sets of nanogels were prepared: **P1** imprinted with the carbonate template **8** (i.e. complex **17**), and **P2** imprinted with the phosphonate template **6** (i.e. complex **18**) (Table 1). All nanogels were fully characterised and were found to be highly soluble in water, **P1**: 9 mg mL^−1^ H_2_O; **P2**: 7 mg mL^−1^ H_2_O, as expected, given their hydrophilic building blocks. Particle sizes were determined in diluted aqueous solutions of the nanogels (0.05 mg polymer mL^−1^) by dynamic light scattering (DLS) (Figure S7). For the carbonate imprinted polymer (**P1**) and the phosphonate imprinted nanogel (**P2**) the particle dimensions were 12±2 nm and 6±1 nm, respectively, measured at 25 °C and 1 mg mL^−1^. The preparations were also shown to have low polydispersity. To determine the incorporation of the monomers into the polymeric matrix and estimate an upper limit for the number of active sites, the cobalt content of the nanogel solutions was analysed by flame atomic absorption spectroscopy (FAAS) and the data showed 36 % incorporation for **P1** and 20 % incorporation for **P2** (Table S1

**Table 1:** Chemical composition and characterisation of MIPs **P1** and **P2**.

Polymer	Template	Acrylamide [%]	EBA [%]	*C*_M_ [%]	Size [nm]	Solubility H_2_O [mg mL^−1^]	% Co content
**P1**	**8**	10	80	0.5	12±2	9	36
**P2**	**6**	10	80	0.5	6±1	7	20

EBA=ethylene bis‐acrylamide; *C*_M_=critical monomer concentration.

To establish whether polymerisation had affected the oxidation state of the cobalt in the polymers imprinted with **6**, nanogel solutions of **P2** were analysed by EPR spectroscopy before and after template removal. The spectra were then compared to the spectrum of the monomer complex **18** (Figure 3 a–c). The EPR data clearly show that the crude **P2** containing the phosphonate template still displays a signal at *g*=4 consistent with the presence of five‐coordinate cobalt(II).^41^ Following template removal, **P2** became EPR‐silent. Complete release of the template was also confirmed by ^31^P NMR spectroscopy and supported the formation of the active Co^III^ aqua‐hydroxo species.

**Figure 4:**
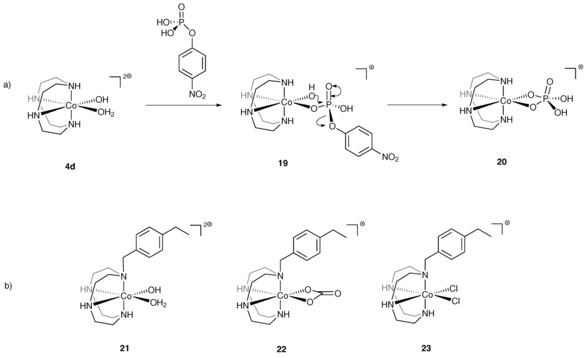
a) Mechanism of phosphate hydrolysis catalysed by **4 d**;^24^ b) non‐polymerisable analogues **21**–**23**.

The first step towards the kinetic studies focused on the evaluation of the hydrolytic activity of **4 d** (Figure 4a) compared to an analogue of the imprinted complexes, as it was previously reported that alkylation at a single nitrogen atom of cobalt(III) cyclen complexes of this type resulted in 25 % reduction in activity.^42^ Complex **21**, a mimic of the active site within the nanogel, was therefore prepared. The most convenient route required the preparation of **22**, which was purified by recrystallization and then converted into **23**, which was again recrystallized. The dichloride species **23** was then converted in situ to the catalytically active complex **21** in HEPES buffer with two equivalents of 0.1 m NaOH. As expected the activity of **21** towards the hydrolysis of **1** was lower, with 67 % of the substrate being hydrolysed after 48 h compared to 75 % for **4 d**, when both complexes were used in excess over the substrate that is, in the complex:substrate ratio 3:1 (Figure S8).

**Figure 5:**
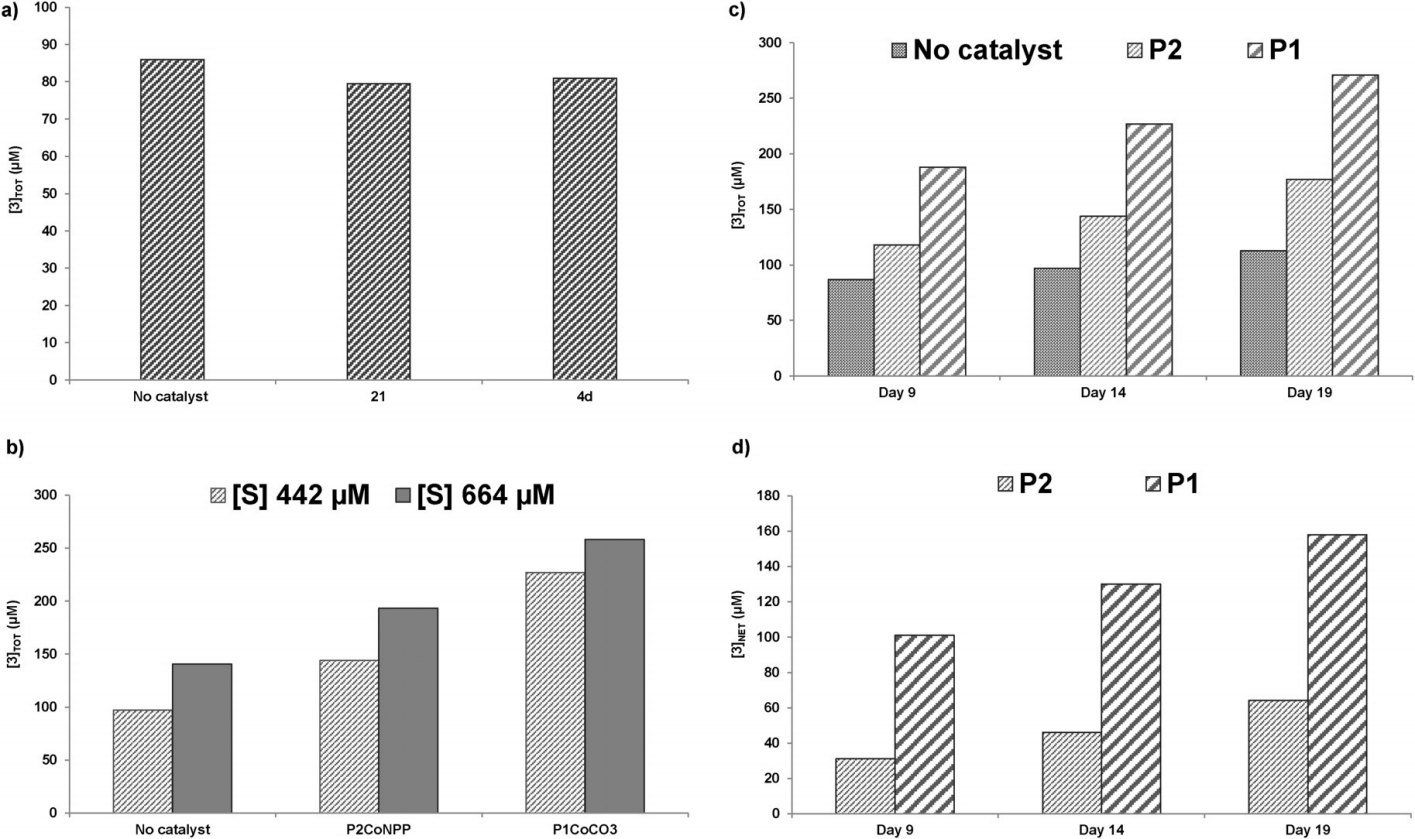
a) Total product **3** (μm) formed after 9 days in the hydrolysis of phosphate **1** (442 μm), without catalyst, catalysed by **21** (100 μm) and by **4 d** (100 μm); b) total product **3** (μm) formed after 14 days in the hydrolysis of **1** (442 μm and 664 μm) without catalyst, catalysed by **P1** (100 μm [Co]) and by **P2** (100 μm [Co]); c) total product **3** (μm) formed after 9, 14 and 19 days in the hydrolysis of **1** (442 μm), without catalyst, catalyzed by **P1** (100 μm [Co]) and by **P2** (100 μm [Co]); d) net product **3** (μm) formed by **P1** (100 μm [Co]) and **P2** (100 μm [Co]) in the hydrolysis of substrate **1** (442 μm) on day 9, 14 and 19; the data have been corrected for the uncatalysed reaction.

However, if the complexes are not used in excess there is no evidence of catalytic activity. Figure 5a shows the total product **3** formed after nine days in the uncatalysed reaction compared to the one catalysed by **4 d** and **21**, respectively. Substrate **1** was kept constant at 442 μm, while both catalysts were used at 100 μm, therefore with a 23 % catalytic load. There is no significant difference between the catalysed reactions and the background, suggesting that product inhibition occurs, a finding consistent with previously reported data for these systems.^43^

The next step focused on the evaluation of the catalytic activity of the cobalt–cyclen nanogels **P1** and **P2**. As the characterisation data clearly indicate (see Table S1 in the Supporting Information), the content of cobalt differs for the two preparations, therefore in order to ensure accurate data, experiments were carried out using different amounts of polymers to ensure equal concentration of active sites. The reactions were carried out under identical experimental conditions, with 442 μm and 664 μm of substrate **1** and 100 μm [Co] for both **P1** and **P2**, which represent 23 % and 15 % catalytic load respectively compared to substrate. Figure 5b shows the total amount of product **3** formed after 14 days with both nanogel preparations compared to the background reaction. It is very clear that both polymers lead to higher product formation, compared to the uncatalysed reaction, at both substrate concentrations. The data also suggest that nanogel **P1** is more efficient. To establish whether this trend was consistent over time, a kinetic experiment was carried out by monitoring total product formation at different time intervals for the background reaction and the ones catalysed by **P1** and **P2**. Figure 5c shows the results obtained at [S]=442 μm, with [Co]=100 μm for both nanogels at day 9, day 14 and day 19. In all three sets of data it can be clearly seen that both nanogels lead to higher product concentration compared to the background reaction, and **P1**, the nanogel imprinted with the carbonate template, consistently performs better than **P2**, imprinted with the cognate template. If the amount of product formed as a result of the background reaction is subtracted from the total product formed, the net product formed as a result of the catalytic complex can be obtained. The data, shown in Figure 5d, clearly demonstrate that not only **P1** is the most efficient polymer but that it operates with turnover, with 130 μm and 158 μm product being formed at 14 and 19 days.

This result is very significant because it demonstrates that the same catalyst, which is required in large excess to accelerate the chemical reaction, when incorporated into a polymeric matrix via the molecular imprinting approach, can be successfully used in catalytic quantities giving turnover. Interestingly, the different activity of the two polymers, used with identical cobalt concentration, can only be the result of morphological changes occurring during the imprinting process as a result of the structures of the templates.

This work demonstrates that the nature of the polymerisable unit and the structure of the template used determine the coordination geometry and oxidation state of the complexes; both pseudo‐octahedral cobalt(III) and trigonal‐bipyramidal cobalt(II) structures can be prepared and their coordination geometry is maintained throughout the polymerisation process. Following the removal of the template, both nanogels contain catalytically active cobalt(III) aqua‐hydroxo species in the cavities. Given the variation in catalytic activity observed, we can deduce that the morphological differences in the polymeric matrices, resulting from the imprinting approach, are responsible for the different molecular recognition capabilities. The nanogel imprinted with the phosphonate in which the metal centre is five‐coordinate increases the activity of the catalyst significantly compared to the free catalyst. It seems plausible that this is the result of the template site disfavouring formation of the inactive post‐hydrolysis complex **20**, Figure 4 a, which is known to inhibit the catalytic cycle. The nanogel imprinted with the carbonate **P1**, shows even higher catalytic efficiency and turnover, despite the carbonate being considerably smaller than the cognate template and containing a pseudo‐octahedral active site. Initially this result appears to be rather counter intuitive and surprising, however, analysis of the crystal structure of an analogue of [Co(**15 b**)(CO_3_)]Cl reveals the O‐Co‐O angle to be rather strained (O(1)‐Co(1)‐O(2)=68.54(9)°),^44^ which is very similar to that seen in other cobalt(III) complexes containing bidentate carbonate ligands in the Cambridge Crystallographic Database.^45^ In contrast, the equivalent angle in cobalt(III) phosphate chelates is considerably larger at 76°.^46^

This suggests that imprinting with carbonate produces an active site in the polymer, which further enhances catalytic activity. A combination of factors is likely to contribute to these results. The first is manifested in both nanogels and is due to the lower polarity within the polymeric matrices, which leads to higher product formation compared to **21**. The second effect results from the significantly more strained coordination geometry for **17**, which results from the tight bite angle of the carbonate ligand; this may cause a difference in stability of the hydrolysis product **2**–polymer complexes, which would then lead to increased activity.

## Conclusion

This work represents the first example of a complex of [Co(cyclen)(OH)(OH_2_)]^2+^, incorporated into a polymeric nanogel with the molecular imprinting approach, that catalyses the hydrolysis of a nerve agent mimic with good turnover, using a low catalytic load (15 %). The increased activity of the polymerically embedded complexes when compared to the free catalyst appears to result from a combination of factors. The local coordination environment at the cobalt centre clearly plays a key role, as evidenced by the significant differences in activity between **P1** and **P2**. However, the fact that both imprinted polymers display catalytic turnover is also likely to be a result of the different local environment within the polymeric nanogel which has a lower polarity compared to bulk aqueous solution; an effect that has been previously observed as an enhancing factor in related systems,4a, 8 Furthermore this study clearly demonstrates the significant variation that can occur in the coordination chemistry and geometry of the active metal ion as result of interactions with structurally similar analogues. These ultimately determine the molecular recognition characteristics and properties of the nanoparticles. In previous reports, in‐depth studies of the coordination geometry of the complex prior to polymerisation have seldom been undertaken,^21^ so that factors that we have shown to occur such as ligand exchange, altered coordination geometry and even oxidation state have not been robustly investigated. These results provide evidence that polymerised biomimetic metal complexes, with well‐defined coordination chemistry, have the potential to deliver viable alternatives when natural proteins are not available.

## Experimental Section

### Materials

All commercially available reagents and solvents were used without further purification, unless otherwise stated. Anhydrous solvents were obtained using an MBraun MB SPS‐800 solvent purification system. To remove traces of water from EtOH, the solvent was dried over 3 Å molecular sieves under N_2_. Distilled water was obtained from an Elga Purelab Option system. Reagents were purchased commercially form Sigma–Aldrich and CheMatech. Nitrogen used for inert atmosphere was oxygen‐free grade. All glassware, glass syringes and metal needles were oven‐dried and cooled prior to experimental use. Infrared spectra were recorded in the range 4000–600 cm^−1^, obtained directly from the compound as a solid on a Bruker Tensor 37 FTIR spectrometer. ^1^H NMR, ^13^C NMR, and ^31^P NMR spectra were recorded using three different NMR instruments: a Jeol JNM‐EX spectrometer (^1^H NMR : 270 MHz; ^13^C NMR: 67.5 MHz; ^31^P NMR: 162 MHz), a Bruker AV400 or a Bruker AMX400 (^1^H NMR : 400 MHz; ^13^C NMR: 100 MHz; ^31^P NMR: 162 MHz). For ^31^P NMR spectra, an external reference spectrum was acquired before each experiment using H_3_PO_4_ (85.0 %) as standard (0.00 ppm). Deuterated solvents and equipment used to record the spectra are stated before each set of data. Chemical shifts are reported in ppm and referenced to residual protonated solvent. Multiplicity is given as follows: s=singlet, d=doublet, t=triplet, q=quartet, dd=doublet of doublets, m=multiplet, bs=broad signal, and coupling constants measured in Hertz (Hz) and reported to 1 d.p.

UV/Vis spectra were obtained on a HP 8453 spectrophotometer, absorption maxima (*λ*_max_) are expressed in nm, the molar extinction coefficients (*ɛ*) are expressed in m ^−1^ cm^−1^. Electrospray ionisation mass spectrometry was carried out by the EPSRC National Mass Spectrometry Service, University of Wales, Swansea on a Thermofisher LTQ Orbitrap XL. Melting points were measured on a Stuart SMP3 melting point apparatus and are uncorrected. Continuous wave (CW) X‐band EPR spectra were obtained at 18±0.2 K using a Bruker ELEXSYS E500 EPR spectrometer equipped with an Oxford Instruments ESR900 helium flow cryostat. Additional experimental parameters were: 0.5 mW microwave power; 100 KHz field modulation; 0.5 mT field modulation. Kinetic studies were carried out by UV/Vis spectroscopy using a HP 8453 spectrophotometer. *p*‐Ethyl benzyl alcohol and *p*‐ethyl benzyl bromide were prepared according to a modified procedure.^31^, ^32^

### Synthesis

**Perhydro‐2 a,4 a,6 a,8 a‐tetraazacyclopenta acenaphthylene (bis‐aminal)**: Cyclen **13** was protected as its bis‐aminal by a slight modification of the reported procedure.^29^ A solution of glyoxal (as a 40 % aqueous solution, 6.00 mL, 52.0 mmol) in MeOH (15.0 mL) was added dropwise at 0 °C to a solution of cyclen (8.00 g, 46.4 mmol) in MeOH (80.0 mL). The mixture was stirred at this temperature for 1 h, heated at 55 °C for a further 2 h, and finally stirred at room temperature for 16 h. The solution was concentrated in vacuo to afford a dark orange oil, which was then triturated with Et_2_O (5×50.0 mL). The organic fractions were combined and the solvent was removed in vacuo to give the bis‐aminal as a white powder (7.43 g, 83 %). All data were consistent with those previously reported. ^1^H NMR (400 MHz, CDCl_3_): *δ*=2.50–2.54 (m, 4 H, CH_2_), 2.65 (bs, 4 H, CH_2_), 2.90–2.99 (m, 8 H, CH_2_), 3.10 ppm (bs, 2 H, CH_aminal_); ^13^C NMR (DEPT 135, 100 MHz, CDCl_3_): *δ*=49.6, 50.4, 76.8 ppm.

**General procedure for mono‐alkyation of the bis‐aminal as demonstrated by the synthesis of 14a (Precursor to 15a) (1*RS*,13*SR*,14*RS*)‐1‐allyl‐4,7,10‐triaza‐1‐azoniatetracyclo tetradecane bromide**: An excess of allyl bromide (R‐X halide) (1.50 mL, 18.0 mmol) was added under an inert atmosphere to a solution of the bis‐aminal (1.75 g, 9.00 mmol) dissolved in the minimum volume of dry toluene (10.0 mL). The mixture was maintained at room temperature for 2 h until a precipitate formed. The resulting white solid was collected by filtration and was washed using freshly distilled toluene (2×5 mL). The collected solids were dried in vacuo to afford the desired product as a white amorphous solid (hygroscopic) (2.90 g, 94 %). The salt exhibits extremely complex ^1^H NMR spectra, as already reported.^30^ ^1^H NMR (400 MHz, D_2_O): *δ*=2.47–2.57 (m, 2 H, CH_2_), 2.77–2.97 (m, 5 H, CH_2_), 3.18–3.34 (m, 4 H, CH_2_), 3.51–3.53 (m, 1 H, CH_2_), 3.61 (bs, 1 H, CH_aminal_), 3.65–3.81 (m, 3 H, CH_2_), 3.96 (bs, 1 H, CH_aminal_), 4.02–4.03 (m, 1 H, CH_2_), 4.12 (dd, *J*=13.1, 5.9 Hz, 1 H, NCH_2_‐C*H*), 4.33 (dd, *J*=13.2, 7.9 Hz, 1 H, NCH_2_‐CH), 5.72–5.78 (m, 2 H, CH=CHH_cis_, CH=CHH_trans_), 6.03–6.13 ppm (m, 1 H, CH=CH_2_); ^13^C NMR (100 MHz, D_2_O): *δ*=43.6, 47.6, 47.8, 48.1, 48.3, 51.2, 57.5, 60.7, 61.6, 71.7, 82.7, 124.2, 129.1 ppm; ESIMS: *m/z* (%): 235 (100) [*M* ^+^]; HRMS (EI) calcd for C_13_H_23_N_4_ [*M*+H]^+^ 235.1917, found 235.1918.

**(1*RS*,13*SR*,14*RS*)‐1‐(4‐Vinylbenzyl)‐4,7,10‐triaza‐1‐azoniatetracyclo tetradecane bromide 14 b (Precursor to 15 b)**: Following the general procedure, bis‐aminal (2.06 g, 10.6 mmol) and 1‐(chloromethyl)‐4‐vinylbenzene (3.00 mL, 21.2 mmol) with a 16 h reaction time at room temperature yielded a white solid (hygroscopic) (3.12 g, 95 %). The salt exhibits complex ^1^H NMR spectra, as already reported.^29^ ^1^H NMR (400 MHz, D_2_O): *δ*=2.49–2.56 (m, 2 H, CH_2_), 2.76–2.88 (m, 3 H, CH_2_), 2.92–2.99 (m, 1 H, CH_2_), 3.07–3.20 (m, 2 H, CH_2_), 3.27–3.37 (m, 3 H, CH_2_), 3.47–3.66 (m, 4 H, CH_2_), 3.75 (d, *J*=4.0 Hz, 1 H, CH_aminal_), 4.02 (d, *J*=4.0 Hz, 1 H, CH_aminal_), 4.19–4.23 (m, 1 H, CH_2_), 4.68–5.87 (m, 2 H, residual HOD solvent peak overlaps signal, N‐CH_2_), 5.44 (d, *J*=11.0 Hz, 1 H, CH=CHH_cis_), 5.96 (d, *J*=17.6 Hz, 1 H, CH=CHH_*trans*_), 6.85 (dd, *J*=11.0, 17.6 Hz, 1 H, CH=CH_2_), 7.57 (d, *J*=8.0 Hz, 2 H, Ar‐H), 7.67 ppm (d, *J*=8.0 Hz, 2 H, Ar‐H); ^13^C NMR (100 MHz, D_2_O): *δ*=43.9, 47.5, 47.6, 48.2, 48.3, 51.2, 57.2, 61.3, 71.7, 82.5, 90.4, 116.5, 126.5, 127.5, 133.2, 135.9, 140.4 ppm; IR: *ν*_max_=2945, 2750, 1620, 1135, 1055 cm^−1^. ESIMS: *m/z* (%): 311 (55) [*M* ^+^], 193 (100), 117 (80); HRMS (EI) calcd for C_19_H_27_N_4_ [*M*+H]^+^ 311.2230, found: 311.2233.

**(4‐Ethylbenzyl)‐4,7,10‐triaza‐1‐azoniatetracyclo tetradecane bromide 14 c (Precursor to 15 c)**: Following general procedure, bis‐aminal (2.00 g, 10.3 mmol) and 4‐ethyl benzyl bromide (2.05 g, 10.3 mmol) with a 20 h reaction time at room temperature yielded a white solid (3.70 g, 91 %). m.p. 165–168 °C. ^1^H NMR (400 MHz, D_2_O): *δ*=1.23 (t, *J*=8.0 Hz, 3 H, CH_3_), 2.48–2.56 (m, 2 H, CH_2_), 2.72 (q_,_ *J*=8.0 Hz, 2 H, CH_2_), 2.77–2.88 (m, 3 H, CH_2_), 2.92–3.00 (m, 1 H, CH_2_), 3.08–3.19 (m, 2 H, CH_2_), 3.25–3.37 (m, 3 H, CH_2_), 3.45–3.77 (m, 4 H, CH_2_), 3.77 (d, *J*=4.0 Hz, 1 H, CH_aminal_), 4.02 (d, *J*=4.0 Hz, 1 H, CH_aminal_), 4.18–4.23 (m, 1 H, CH_2_), 4.68–4.86 (m, 2 H, residual HOD solvent peak overlaps signal, N‐CH_2_), 7.44 (d, *J*=8.0 Hz, 2 H, Ar‐H), 7.51 ppm (d, *J*=8.0 Hz, 2 H, Ar‐H); ^13^C NMR (100 MHz, D_2_O): *δ*=14.7, 28.0, 43.8, 47.5, 48.3, 51.3, 57.2, 61.2, 61.4, 62.5, 71.7, 82.3, 90.5, 124.0, 129.0, 132.5, 148.1 ppm; IR: *ν*_max_=2961, 2883, 2848, 1612, 1514, 1433, 1266, 1219, 1184, 1134, 1053, 1027, 982, 929, 846 cm^−1^; HRMS (EI) calcd for C_19_H_29_N_4_ [*M*+H]^+^ 313.2387, found: 313.2383.

**1‐Allyl‐1,4,7,10‐tetraazacyclododecane 15 a**:^33^ Compound **14 a** (0.385 g, 1.22 mmol) was dissolved in dry EtOH (6 mL). Hydroxylamine degassed in a 50 % aqueous solution (0.360 mL, 12.2 mmol) was added dropwise to this solution and the resulting mixture was stirred for 20 min at room temperature and then for 120 min at about 50 °C, making the solution turn from cloudy white to clear pale yellow. After cooling, the reaction mixture was stirred for a further hour at room temperature before an equal volume of 10 % w/w KOH was added. The mixture was extracted with DCM (5×10.0 mL) and the organic fractions were combined, dried over MgSO_4_, and removed in vacuo to afford the product as a pale yellow oil (0.190 g, 73 %). ^1^H NMR (270 MHz, CDCl_3_): *δ*=2.50–2.61 (m, 12 H, CH_2_), 2.71–2.75 (m, 4 H, CH_2_), 3.06 (d, *J*=6.5 Hz, 2 H, NCH_2_‐CH), 5.06–5.13 (m, 2 H, CH=CHH_cis_, CH=CHH_trans_), 5.79 ppm (m, 1 H, CH=CH_2_); ^13^C NMR (67.5 MHz, D_2_O): *δ*=44.8, 45.2, 45.8, 46.8, 50.8, 51.3, 57.4, 117.4, 135.3 ppm; IR (CHCl_2_, NaCl): *ν*_max_=3658, 3250, 3053, 2961, 2852, 2305, 1451, 1421, 1265, 734 cm^−1^.

**1‐(4‐Vinylbenzyl)‐1,4,7,10‐tetraazacyclododecane 15 b**:^33^ Compound **14 b** (0.500 g, 1.44 mmol) was dissolved in dry EtOH (6 mL). Hydroxylamine in a 50 % aqueous solution (0.420 mL, 14.4 mmol) was added dropwise to this solution and the resulting mixture was stirred for 20 min at room temperature and then for 16 h at about 60 °C, making the solution turn from cloudy white to clear pale yellow. After cooling, the reaction mixture was stirred for a further hour at room temperature before an equal volume of 10 % w/w KOH was added. The mixture was extracted with CHCl_3_ (5×10 mL) and the organic fractions were combined, dried over MgSO_4_, and removed in vacuo to afford the crude product as a yellow oil (0.400 g, 96 %). This crude product contains some minor impurities and required further purification. The crude oil was suspended in EtOH (1 mL), and HCl (35 m, 2 mL) was added dropwise to obtain a hydrochloride salt of **15 b** as a white precipitate. This precipitate was filtered, dissolved in a solution of KOH (40 % aqueous solution, w/v, 10 mL), and extracted with CHCl_3_ (5×30 mL). The organic layers were combined, dried over MgSO_4_, and removed in vacuo to afford **15 b** as a yellow oil (60.0 mg,). ^1^H NMR (400 MHz, CDCl_3_): *δ*=2.58–2.83 (m, 16 H, CH_2_), 3.61 (s, 2 H, N‐CH_2_), 5.18 (d, *J*=10.9 Hz, 1 H, CH=CHH_cis_), 5.69 (d, *J*=17.7 Hz, 1 H, CH=CHH_*trans*_), 6.67 (dd, *J*=10.9, 17.7 Hz, 1 H, CH=CH_2_), 7.25 (d, residual solvent peak overlaps signal*, J*=8.0 Hz, 2 H, Ar‐H), 7.34 ppm (d, *J*=8.0 Hz, 2 H, Ar‐H); ^13^C NMR (100 MHz, CDCl_3_): *δ*=45.2, 46.1, 47.2, 51.3, 59.2, 113.5, 126.2, 129.3, 136.9, 138.3, 146.3 ppm; IR: *ν*_max_=2807, 2363, 1628, 1567, 1450, 1348, 1263 cm^−1^.

**1‐(4‐Ethylbenzyl)‐1,4,7,10‐tetraazacyclododecane 15 c**: Compound **14 c** (0.800 g, 2.03 mmol) was dissolved in dry EtOH (10 mL). Hydroxylamine in a 50 % aqueous solution (0.550 mL, 20.3 mmol) was added dropwise to this solution and the resulting mixture was stirred for 20 min at room temperature and then for 16 h at about 60 °C, making the solution turn from cloudy white to clear pale yellow. After cooling, the reaction mixture was stirred for a further hour at room temperature. The reaction mixture was then reduced in volume to afford a yellow oil, which was dissolved in HCl (5.00 m, 2 mL). The aqueous layer was washed with EtOAc (20 mL × 2) and Et_2_O (20 mL × 2). The resulting aqueous layer was then adjusted to pH 14.0 with KOH (40 % aqueous solution, w/v, 2 mL) and extracted with CHCl_3_ (5×30 mL). Organic fractions were then combined, dried over MgSO_4_, and removed in vacuo to afford the crude product as a yellow oil. After suspending the oil in EtOH (1 mL), HCl (35 m, 2 mL) was added dropwise to obtain a hydrochloride salt of **15 c** as a white precipitate. This precipitate was collected by filtration, dissolved in a solution of KOH (40 % aqueous solution, w/v, 10 mL), and extracted with CHCl_3_ (5×30 mL). The organic layers were combined, dried over MgSO_4_, and concentrated in vacuo to afford **15 c** as a yellow oil (0.106 g, 18 %). ^1^H NMR (400 MHz, CDCl_3_): *δ*=1.21 (t, *J*=8.0 Hz, 3 H, CH_3_), 2.58–2.69 (m, 14 H, CH_2_), 2.81–2.83 (m, 4 H, CH_2_), 3.59 (s, 2 H, N‐CH_2_), 7.13 (d, *J*=8.0 Hz, 2 H, Ar‐H), 7.20 ppm (d, *J*=8.0 Hz, 2 H, Ar‐H); ^13^C NMR (100 MHz, CDCl_3_): *δ*=15.6, 28.6, 45.5, 46.4, 47.6, 51.3, 59.2, 127.9, 129.1, 136.2, 143.1 ppm; IR: *ν*_max_=3280, 2928, 1671, 1511, 1455, 1349, 1263, 1113, 1041, 931, 818 cm^−1^; HRMS (EI) calcd for C_17_H_31_N_4_ [*M*+H]^+^ 291.2543, found 291.2542.

**General procedure for the synthesis of Co^III^CO_3_ complexes, as demonstrated by the synthesis of** ***cis*** **‐[Co(cyclen)CO_3_]HCO_3_ 5**:^[47]^ Cyclen **13** (0.193 g, 0.550 mmol) was dissolved in a MeOH:H_2_O mixture (1:1) (6 mL) and an equimolar amount of Na_3_[Co(CO_3_)_3_]⋅3 H_2_O (0.200 g, 0.550 mmol) was added. The dark green solution gradually turned burgundy‐red and was left to react for 16 h at 65 °C. The solution was filtered whilst hot under suction to separate the liquid from a black solid. The filtrate was dried in vacuo, redissolved in MeOH (6 mL), and the resulting solution was filtered to remove white precipitate. The resulting filtrate was reduced in volume in vacuo and excess Et_2_O (30 mL) was added. The resulting solids were collected by filtration and washed with Et_2_O (10 mL) to afford **5** as a pink powder (0.170 g, 87 %). ^1^H NMR (400 MHz, D_2_O): *δ*=2.47–3.20 (m, 14 H, CH_2_), 3.55–3.62 ppm (m, 2 H, CH_2_); IR: *ν*_max_=3157, 3080, 2959, 2896, 1619, 1440, 1405, 1346, 1242, 834 cm^−1^; UV/Vis (H_2_O): *λ*_max_ (*ɛ*)=525 (271), 368 nm (200 dm^3^ mol^−1^ cm^−1^).

***cis*‐[Co(15 a)CO_3_]HCO_3_ 16**: Following the general procedure, cyclen derivative **15 a** (0.161 g, 0.757 mmol) and Na_3_[Co(CO_3_)_3_]⋅3 H_2_O (0.274 g, 0.757 mmol) with a reaction time of 16 h yielded complex **16** as a dark red powder (89.0 mg, 30 %). ^1^H NMR (400 MHz, D_2_O): *δ*=2.61–3.49 (m, 18 H, CH_2_, N‐CH_2_), 5.48 (m, 2 H, CH=CH_2_), 6.09 ppm (dd, *J*=9.8, 17.7 Hz, 1 H, CH‐CH_2_); UV/Vis (H_2_O): *λ*_max_ (*ɛ*)=536 (215), 350 nm (213 dm^3^ mol^−1^ cm^−1^); ESI *m/z* (%): 331.2 (100) [*M* ^+^], 269.2 (30); HRMS (ESI): calcd for C_12_H_24_O_3_N_4_Co [*M*+H^+^] 331.1175, found: 331.1175.

***cis*‐[Co(15 b)CO_3_]HCO_3_ 17**: Following the general procedure, cyclen derivative **15 b** (0.216 g, 0.750 mmol) and Na_3_[Co(CO_3_)_3_]⋅3 H_2_O (0.271 g, 0.750 mmol) with a reaction time of 16 h yielded complex **17** as a burgundy red powder (0.237 g, 67 %). ^1^H NMR (400 MHz, D_2_O): *δ*=2.60–2.76 (m, 3 H, CH_2_), 2.85‐3.23, (m, 12 H, CH_2_), 3.40‐3.45 (m, 2 H, CH_2_), 3.83 (d, *J_AB_*=12.0 Hz, 1 H, N‐CH_2_), 4.00 (d, *J_AB_*=12.0 Hz, 1 H, N‐CH_2_), 5.39 (d, *J*=11.0 Hz, 1 H, CH=CHH_cis_), 5.92 (d, *J*=17.7 Hz, 1 H, CH=CHH_*trans*_), 6.84 (dd, *J*=11.0, 17.7 Hz, 1 H, CH=CH_2_) 7.44 (d, *J*=8.0 Hz, 2 H, Ar‐H), 7.58 ppm (d, *J*=8.0 Hz, 2 H, Ar‐H). IR: *ν*_max_ 3089, 2890, 1613, 1449, 1344, 1291, 828, 750 cm^−1^; UV/Vis (DMSO): *λ*_max_ (*ɛ*)=539 nm (213 dm^3^ mol^−1^ cm^−1^); ESIMS *m/z* (%): 407.1 (43) [*M* ^+^], 351.2 (55), 701.4 (100); HRMS (EI) calcd for C_18_H_28_O_3_N_4_Co−HCO_3_ [*M*+H]^+^ 407.1488, found 407.1487.

***cis*‐[Co(15 c)CO_3_]HCO_3_ 22**: Following the general procedure, cyclen derivative **15 c** (95.0 mg, 0.330 mmol) and Na_3_[Co(CO_3_)_3_]⋅3 H_2_O (0.120 g, 0.330 mmol) with a reaction time of 16 h yielded complex **22** as a dark pink powder (0.125 g, 81 %). m.p. 184–186 °C. ^1^H NMR (400 MHz, D_2_O): *δ*=1.23 (t, *J*=8.0 Hz, 3 H, CH_3_), 2.58–2.77 (m, 6 H, CH_2_), 2.86–3.23 (m, 10 H, CH_2_), 3.44–3.46 (m, 2 H, CH_2_), 3.84 (d, *J_AB_*=16.0 Hz, 1 H, N‐CH_2_), 3.98 (d, *J_AB_*=12.0 Hz, 1 H, N‐CH_2_), 7.39 ppm (m, 4 H, Ar‐H); ^13^C NMR (100 MHz, D_2_O): *δ*=14.9, 27.9, 47.1, 47.5, 48.4, 49.4, 53.1, 55.6, 55.8, 56.9, 63.1, 126.9, 128.2, 132.6, 146.3, 167.0 ppm; IR: *ν*_max_=3379, 3112, 2882, 1957, 1450, 1337, 1264, 1058, 1016, 976, 828, 750 cm^−1^; UV/Vis (HPLC MeOH): *λ*_max_ (*ɛ*)=366 (472), 540 nm (542 dm^3^ mol^−1^ cm^−1^); HRMS (EI) calcd for C_18_H_30_CoN_4_O_3_ [*M*+H]^+^ 409.1644, found 409.1636.

**General procedure for the synthesis of Co^III^Cl_2_ complexes as demonstrated by the synthesis of** ***cis*** **‐[Co(cyclen)Cl_2_]Cl 4 a**:^[34]^ Complex **5** (0.205 g, 0.580 mmol) was dissolved in MeOH (8 mL) and HCl (35 m, 2 mL) was added dropwise to this solution. The reaction mixture was reduced to dryness and the residue was again dissolved in MeOH (8 mL) and treated with HCl (35 m, 2 mL). This procedure was repeated five times and a gradual colour change from dark pink to dark violet was observed. The final solution was concentrated in vacuo and the resulting violet solid was washed with Et_2_O (20 mL) to afford **4 a** as a violet crystalline solid (0.220 g, 75 %). ^1^H NMR (400 MHz, [D_6_]DMSO): *δ*=2.25–3.45 (m, 16 H, CH_2_), 7.48 (bs, 1 H, NH), 7.59 (bs, 1 H, NH), 7.95 ppm (bs, 1 H, NH); ^13^C NMR (100 MHz, [D_6_]DMSO): *δ*=46.3, 49.7, 54.3, 57.5 ppm; IR: *ν*_max_=3210, 3172, 3050, 2870, 1645, 1477, 1444, 1356, 1112, 1057 cm^−1^; UV/Vis (DMSO): *λ*_max_ (*ɛ*)=561 (134), 390 nm (53 dm^3^ mol^−1^ cm^−1^); ESIMS: *m/z* (%): 301.0 (100) [*M* ^+^], 358.9 (15), 545.4 (25); HRMS (EI) calcd for C_8_H_20_N_4_CoCl_2_ [*M*+H]^+^ 301.0392, found 301.0392.

***cis*‐[Co(15 c)Cl_2_]Cl 21 b**: Following the general procedure, carbonate complex **22** (0.120 g, 0.250 mmol) yielded complex **23** as a dark violet solid (0.129 g, 86 %). m.p. 195–198 °C; ^1^H NMR (400 MHz, MeOD): *δ*=1.23 (t, *J*=8.0 Hz, 3 H, CH_3_), 2.52–2.73 (m, 9 H, CH_2_), 3.32–3.79 (m, 16 H, CH_2_, residual MeOD proton peak overlaps signal), 4.52 (dd, *J_AB_*=4.0 Hz, 16.0 Hz, 2 H, N‐CH_2_), 7.27 (d, *J*=4.0 Hz, 2 H, Ar‐H), 7.32 ppm (d, *J*=8.0 Hz, 2 H, Ar‐H); ^13^C NMR (100 MHz, D_2_O): *δ*=16.0, 29.5, 51.0, 51.2, 50.2, 58.3, 59.4, 64.0, 65.1, 129.4, 130.3, 133.5, 146.6 ppm; IR: *ν*_max_ =3056, 2962, 2870, 1613, 1447, 1098, 1063, 1008, 970, 828 cm^−1^; UV/Vis (MeOH): *λ*_max_ (*ɛ*)=570 (224), 395 nm (264 dm^3^ mol^−1^ cm^−1^); HRMS (EI) calcd for C_17_H_28_CoN_4_ [*M*+H]^+^ 347.1640, found 347.1639.

**4‐Nitrophenyl phosphonate (6)**:^48^ An excess of bromotrimethylsilane (4.50 mL, 34.1 mmol) was added dropwise at 0 °C to a solution of diethyl 4‐nitrobenzylphosphonate (3.30 mL, 15.0 mmol) in acetonitrile (20 mL). The ice bath was removed and the mixture was stirred for 24 h at room temperature. The resulting dark yellow solution was concentrated in vacuo and the residue was dissolved in MeOH (20 mL). Stirring was maintained for a further 24 h at room temperature, after which time the solvent was removed in vacuo, leaving a beige solid. The solid was repeatedly washed with DCM (30 mL) and then dried in vacuo to give **6** as an off‐white solid (3.00 g, 92 %). m.p. 224–226 °C; ^1^H NMR (400 MHz, [D_6_]DMSO): *δ*=3.16 (d, *J*=22.0 Hz, 2 H, CH_2_), 7.51 (dd, *J*=8.8, 2.3 Hz, 2 H, Ar‐H), 8.20 (d, *J*=8.3 Hz, 2 H, Ar‐H), 9.75 ppm (bs, 2 H, OH); ^13^C NMR (100 MHz, D_2_O): *δ*=35.1, 123.7, 130.4, 142.1, 146.3 ppm; ^31^P NMR (161 MHz, [D_6_]DMSO): *δ*=19.37 ppm; ^31^P NMR (161 MHz, CD_3_OD): *δ*=22.07 ppm; ^31^P NMR (161 MHz, D_2_O): *δ*=22.38 ppm; IR: *ν*_max_ =2613, 1599, 1520, 1344, 1268, 944, 771, 695 cm^−1^.

**General procedure for the synthesis of phosphonate complexes as demonstrated by the synthesis of** ***cis*** **‐[Co(cyclen)NPPA]Cl 9**: Cyclen **13** (48.2 mg, 0.280 mmol) was dissolved in degassed MeOH (2 mL), and an equimolar solution of CoCl_2_⋅6 H_2_O (66.4 mg, 0.280 mmol) in MeOH (5 mL) was added under N_2_, causing the solution to turn brown and shortly after pale violet. After stirring the mixture for 5 min, an equimolar solution of the phosphonate **6** (60.5 mg, 0.280 mmol) in MeOH (5 mL) was added under an inert atmosphere, and the solution turned to dark purple. Stirring under N_2_ was maintained for 5 h at room temperature, after which the solution was bubbled with compressed air for an equal amount of time. The solvent was removed in vacuo and the resultant solids were collected by filtration and washed with Et_2_O (10 mL), yielding **9** as a purple solid (108 mg, 84 %). m.p. 240 °C (decomposes); ^31^P NMR (161 MHz, D_2_O): *δ*=32.1 ppm. IR: *ν*_max_=2855, 1597, 1511, 1452, 1343, 1237, 1058, 1007, 918, 858, 813, 768, 727, 695 cm^−1^; UV/Vis (DMSO): *λ*_max_ (*ɛ*)=548 nm (160 dm^3^ mol^−1^ cm^−1^); ESIMS: *m/z* (%): 446.1 (100) [*M* ^+^], 663.1 (25); HRMS (EI) calcd for C_15_H_26_N_5_O_5_PCo [*M*+H]^+^ 446.1015, found 446.098.

***cis*‐[Co(cyclen)(7)]Cl 10**: Following the general procedure, cyclen **13** (29.2 mg, 0.170 mmol) with phosphonate **7** (26.9 mg, 0.170 mmol) and CoCl_2_⋅6 H_2_O (40.3 mg, 0.170 mmol) yielded **10** as a purple solid (50.0 mg, 68 %). m.p. 224–225 °C; ^31^P NMR (161 MHz, D_2_O): *δ*=32.1 ppm. IR: *ν*_max_ =2858, 1638, 1479, 1437, 1134, 1057, 1005, 920, 752, 697, 616, 810, 712 cm^−1^; UV/Vis (H_2_O): *λ*_max_ (*ɛ*)=372 (2126), 532 nm (307 dm^3^ mol^−1^ cm^−1^); UV/Vis (DMSO): *λ*_max_ (*ɛ*)=529 nm (155 dm^3^ mol^−1^ cm^−1^); ESI: *m/z* (%): 387.1 (100) [*M* ^+^]; HRMS (EI) calcd for C_14_H_25_N_4_O_3_CoP [*M*+H]^+^ 387.1007, found 387.0989.

***cis*‐[Co(15 b)(6)]Cl 18**: Following the general procedure, cyclen derivative **15 b** (0.484 g, 1.68 mmol) with phosphonate **6** (0.363 g, 1.68 mmol) and CoCl_2_⋅6 H_2_O (0.398 g, 1.68 mmol) yielded **18** as a blue‐green powder (0.869 g, 91 %). This procedure results in the reproducible formation of the same mixture of compounds and the data presented should be regarded as being diagnostic, rather than corresponding to a single species with complete bulk purity. m.p. 120–125 °C. IR: *ν*_max_ =1601, 1513, 1344, 1247, 1153, 1109, 1035, 919, 858, 772, 729, 696 cm^−1^; UV/Vis (DMSO): *λ*_max_ (*ɛ*)=556 (78), 602 (91), 615 (90), 655 nm (84 dm^3^ mol^−1^ cm^−1^); ESIMS: *m/z* (%): 737.4 (40) [*M* ^+^], 562.2 (50), 551.2 (100); HRMS (EI) cacld. for C_24_H_34_O_5_N_5_CoP [*M*+H]^+^ 562.1624, found 562.1569.

### EPR spectroscopy

EPR spectra were recorded at X‐band (approximately 9.4 GHz on the spectrometer employed) using a Bruker ELEXSYS E500/E580 spectrometer. Temperature control was provided by an Oxford Instruments ESR900 liquid helium cryostat and an ITC503 temperature controller. The spectra were recorded at the Manchester Interdisciplinary Biocentre, University of Manchester (UK).

### Cobalt content characterisation by atomic absorption (FAAS)

Solutions of nanogels (0.30 mg mL^−1^) in at least 3.00 mL of water were prepared and tested for Co absorbance against a set of five cobalt standard calibration solutions (1.00–5.00 μg mL^−1^), showing absorbance values in the 2.00–4.00 μg mL^−1^ range. The amount of cobalt present in solution was taken from the absorbance values, which were converted into concentration using the Beer–Lambert Law. The yield of metallomonomer incorporated was calculated by dividing the amount of cobalt found by atomic absorption by the theoretical amount initially introduced into the polymer mixture.

## Supporting information

As a service to our authors and readers, this journal provides supporting information supplied by the authors. Such materials are peer reviewed and may be re‐organized for online delivery, but are not copy‐edited or typeset. Technical support issues arising from supporting information (other than missing files) should be addressed to the authors.

Supplementary
