# Supplementary material for: Incorporation of Cobalt‐Cyclen Complexes into Templated Nanogels Results in Enhanced Activity
Source: Chemistry. 2015 Dec 10;22(11):3764–74. doi: 10.1002/chem.201503946 (PMC4797703; doi:10.1002/chem.201503946)
Supplement: Supplementary file 1 — Supplementary [file CHEM-22-3764-s001.pdf]

CHEMISTRY
A European Journal

Supporting Information

Incorporation of Cobalt-Cyclen Complexes into Templated Nanogels Results in Enhanced Activity

Ana Rita Jorge,[a] Mariya Chernobryva,[a] Stephen E. J. Rigby,[b] Michael Watkinson,*[a] and Marina Resmini*[a]

chem_201503946_sm_miscellaneous_information.pdf

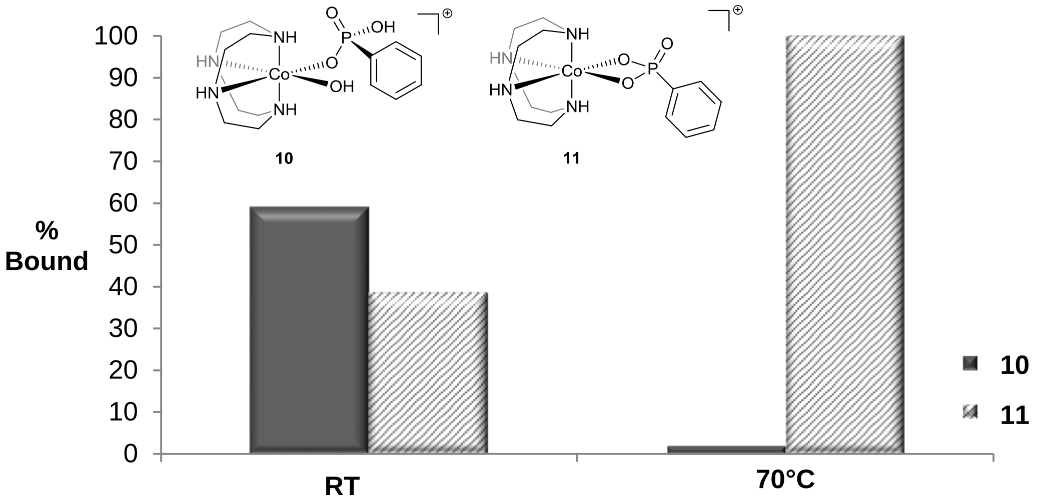

Fig. S1 Study of the interaction between free complex **4a** and phosphonate template **7** at different temperatures in aqueous solution at pD 8.7

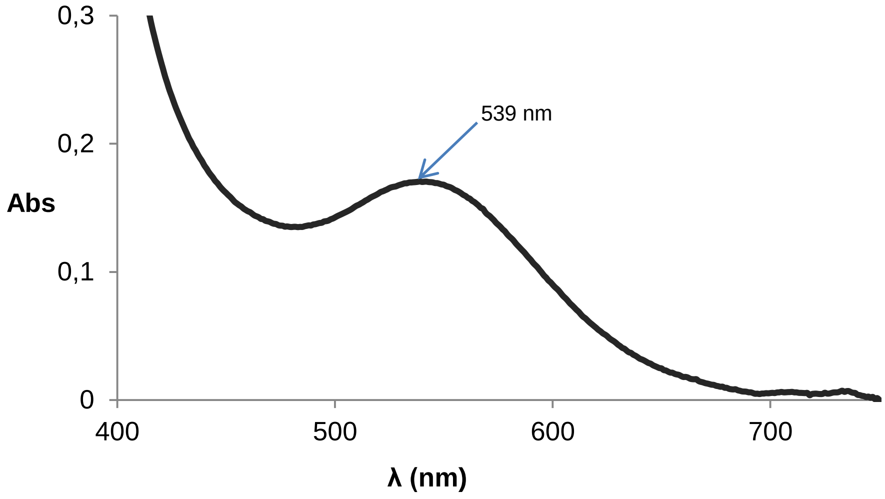

Fig. S2 UV-Vis spectrum of carbonate complex **17** (0.8 mM DMSO solution).

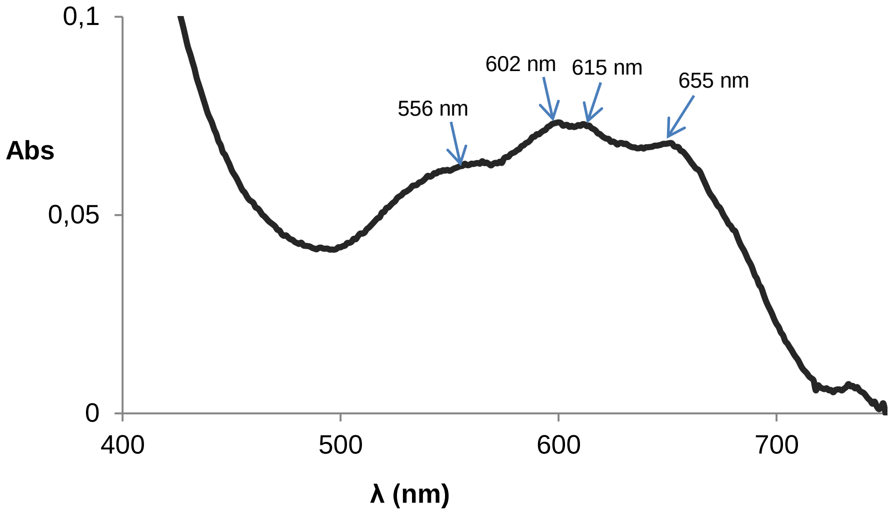

Fig. S3 UV-Vis spectrum of the phosphonate complex **18** (0.8 mM DMSO solution).

EPR experiments

EPR spectra were recorded at X-band (approximately 9.4 GHz on the spectrometer employed) using a Bruker ELEXSYS E500/E580 spectrometer. Temperature control was provided by an Oxford Instruments ESR900 liquid helium cryostat and an ITC503 temperature controller. The spectra were recorded by Dr. Steven Rigby at the Manchester Interdisciplinary Biocentre, University of Manchester (U.K.).

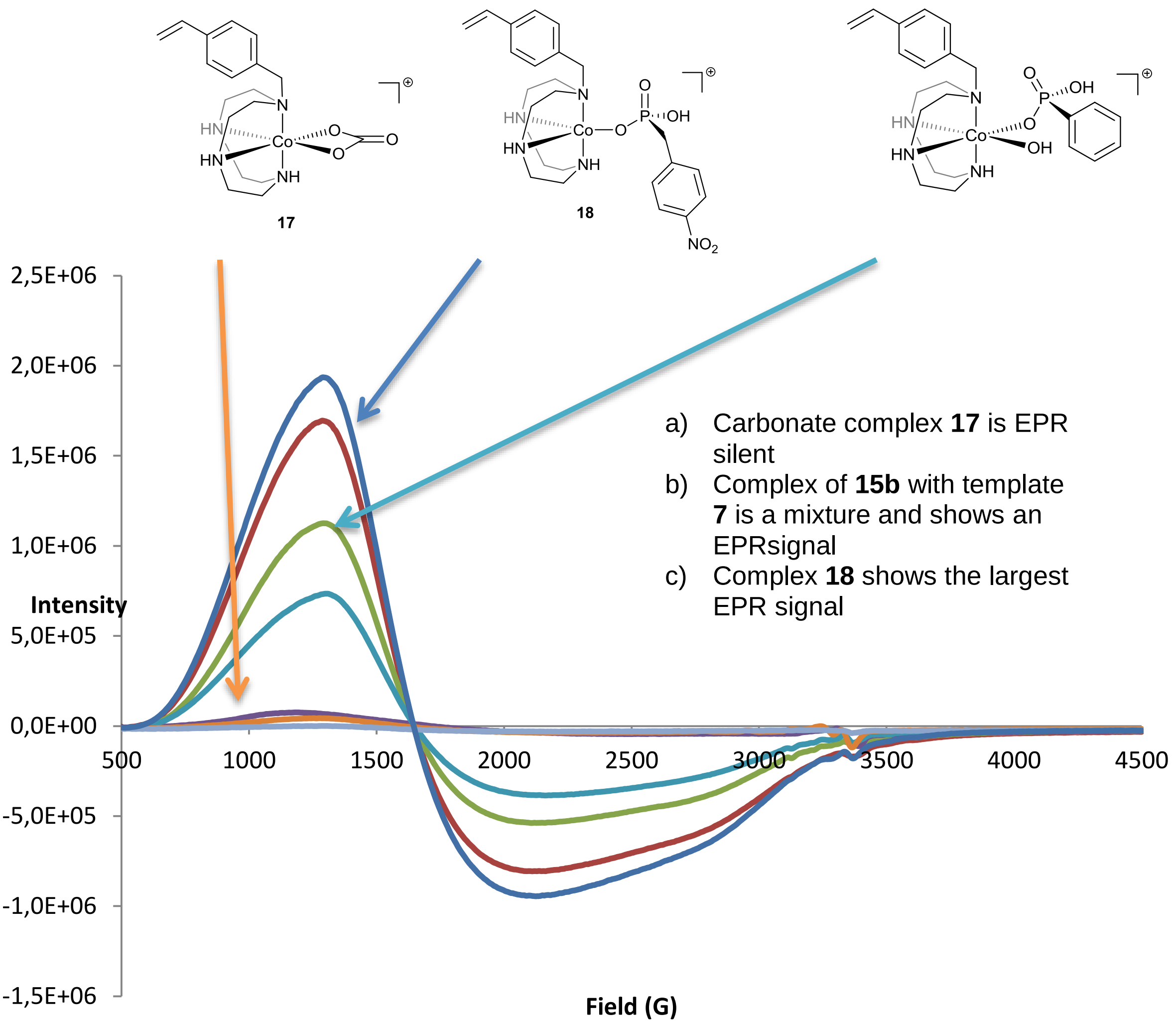

Fig. S4 EPR spectra of frozen solutions (3 mM DMSO) of polymerisable complexes **17**, **18** and the complex of **15b** and template **7**.

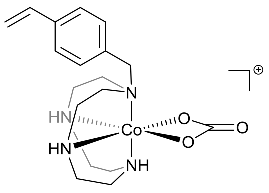

17

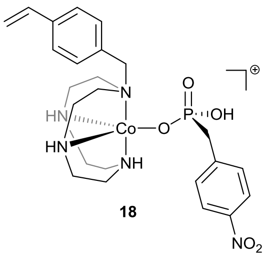

18

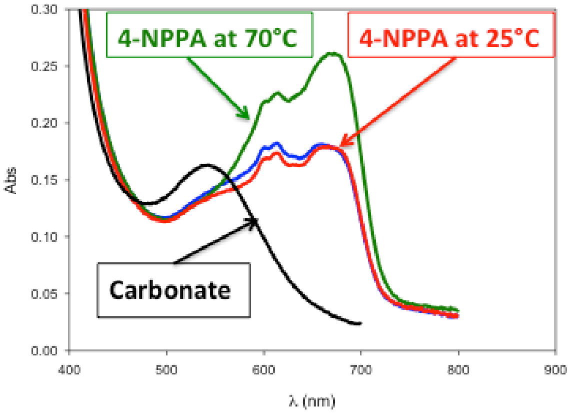

Fig. S5 Study of the stability of the complexes **17** and **18** at different temperatures: the blue line shows that the complex returns to its original spectrum when the temperature is back to 25ºC.

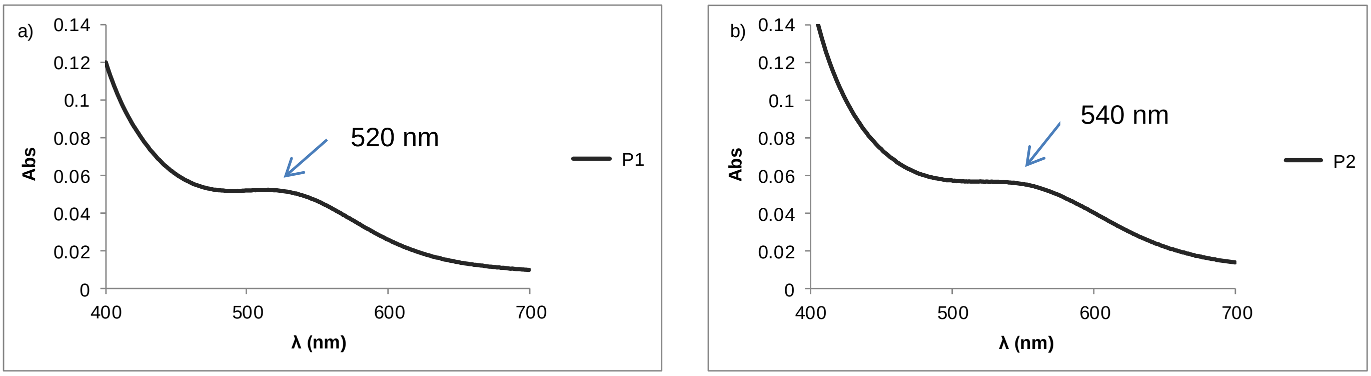

Fig. S6 UV-visible spectra of **P1** (a) and **P2** (b) (100 µM) in HEPES (10 mM, pH 7.2) pH following template removal.

Synthesis

Sodium tris-carbonatocobaltate (III).trihydrate.[1] A solution of commercial H_2O_2 (30% aqueous solution (v/v), (0.580 mL) was added to an aqueous solution (2.50 mL) of $Co(NO_3)_2.6H_2O$ (1.45 g, 5.00 mmol) . The mixture was added dropwise to a stirred slurry of $NaHCO_3$ (2.10 g, 25.0 mmol) in distilled water (5.00 mL) at 0 °C, and precipitation occurred immediately. The mixture was stirred for 1 h at 0 °C, after which time the green solid product was collected by filtration and washed with equal volumes of cold water (5.00 mL), absolute EtOH and Et_2O. The resulting olive green solid was dried in air for 4 days to yield the pure salt (1.22 g, 67%). IR: (ν_{max}/cm^{-1}) 3218, 1640, 1454, 1354, 832.

Synthesis of the Nanogels

General Procedure

The preparation of the imprinted nanogels was started by weighing complex **17** (172 mg, 0.370 mmol) or **18** (220 mg, 0.370 mmol), acrylamide (26.3 mg, 0.370 mmol) and *N,N*-ethylenebisacrylamide (497 mg, 2.96 mmol). Recrystallised AIBN was used as the initiator, in 2% of the overall mass of double bonds in the monomers (22.0 mg, 0.133 mmol). The mixture of monomers and initiator was dissolved in DMSO (130 mL or 138 mL), in an amount that ensured that the overall mass of monomers made up 0.5% of the overall weight of the solution, according to the Eq. S1. The polymer solution was maintained at 70 °C for 4 days under an inert atmosphere. Subsequently the solution was cooled to RT, transferred to a hydrated dialysis membrane (3500 kDa cut-off). Dialysis was performed for 4 days against a diluted solution of HCl (pH 4.5), with frequent changes of this solution The dialysed solution was then transferred to a round bottomed flask where HCl 1 M (3 mL) was added in an ice bath (0 °C) and dialysis was repeated. The nanogel solution was then freeze dried to give the nanogels **P1** and **P2** in 16% and 19% yields respectively as light pink fluffy powders.
A control polymer was also prepared under the same conditions used for the imprinted nanogels. The control did not contain any metallomonomers but contained the same amount of crosslinker and initiator which allowed a comparison between polymer yields in the presence and absence of metals to be made. Acrylamide was present in double the amount to compensate for the lack of functional monomer in solution. Control nanogel **P0** was obtained in 81% yield as a white fluffy powder.

$$C_M = \frac{W_{monomers}}{W_{solution}} * 100 \qquad \textbf{(Eq. S1)}$$

Where:

- $W_{monomers} = W_{complex} + W_{acrylamide} + W_{cross\text{-}linker} + W_{initiator}$
- $W_{solution} = W_{monomers} + W_{solvent}$
- $mol_{initiator} = [(mol_{complex} \times 1_{double\ bond}) + (mol_{acrylamide} \times 1) + (mol_{crosslinker} \times 2)]*0.02$

Solubility

Solubility was tested by weighing the polymer accurately and adding increasing amounts of solvent until the solutions became transparent and no particles were visibly seen in suspension. The minimum weight of solvent necessary for the colloidal stability of the particles was recorded and transformed into volume.

Size Characterisation by DLS using a Zetasizer Nano

The nanogels were dissolved in HEPES buffer (10 mM, pH 7.2) (or DMSO) in low concentration (0.05 mg/mL). The solutions were then filtered into a cuvette using a PTFE filter with a pore size of 1 µm, in order to eliminate any dust particles and aggregates, present in solution. Size distribution was obtained as a function of the intensity of scattered light using the refractive index of water and DMSO (H_2O: n_D^{25} 1.33; DMSO: n_D^{25} 1.48). Size distribution as a function of volume and number of particles was determined by adding the refractive index of polymethacrylate (n_D^{25} 1.4914 and dn/dλ -0.0575).

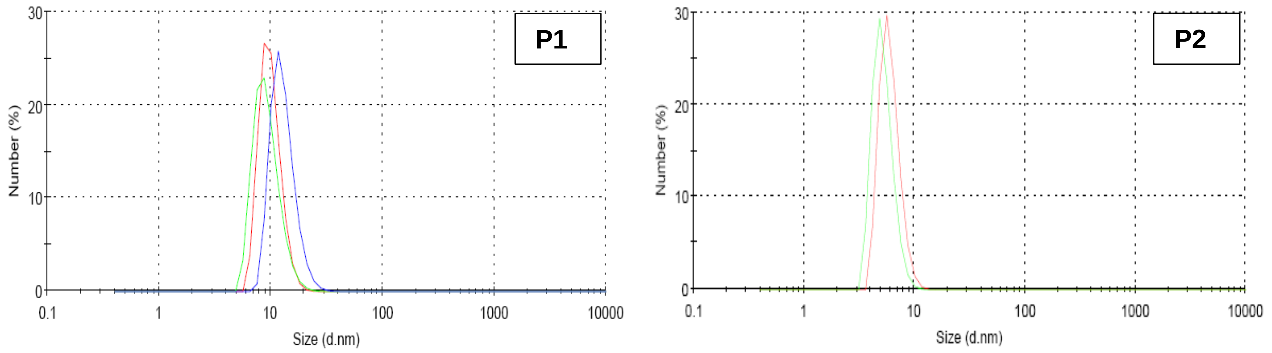

Fig. S7 Size distribution (by number) for an aqueous solution of **P1** and **P2** (C=0.05 mg/mL).

Cobalt Content Characterisation by Atomic Absorption (FAAS)

Solutions of nanogels (0.30 mg/mL) in at least 3.00 mL of water were prepared and tested for Co absorbance against a set of 5 cobalt standard calibration solutions (1.00 – 5.00 μg/mL), showing absorbance values in the 2.00 – 4.00 μg/mL range. The amount of cobalt present in solution was taken from the absorbance values, which were converted into concentration using the Beer-Lambert Law. The yield of metallomonomer incorporated was calculated by dividing the amount of cobalt found by atomic absorption by the theoretical amount initially introduced into the polymer mixture.

	Polymer	Polymerisable unit	Abs (a.u.)	[Cobalt] by FAAS (μg/mL)	[sample] (mg $_{polymer}$/mL)	Theoretical Co content (μg/mL)	Co incorporation (%)
**P0**	**Control**	n/a	0.001	0.000	0.300	0.000	0.0%
**P1**	**MIP-CO_3**	Styrene	0.170	3.325	0.300	9.140	36.0%
**P2**	**MIP-NPPA**	Styrene	0.096	1.860	0.300	9.250	20.0%

Table S1. Incorporation of the cobalt metallomonomers in the nanogels (given as %). **P0** given as a blank.

Cleavage and Rebinding of Template

To test for the presence of template in the nanogels, a solution of polymer was dissolved in D_2O (5.00 mg/mL) and DCl (0.1 ml 1.00 M) was added. The solutions were evaluated by ^{31}P NMR to detect the presence of signals in the free phosphonate region (dependent on template used). For the rebinding experiments, a solution of nitrophenyl phosphonic acid in HEPES (10.0 mM in D_2O) was added to a solution of (P1) (2.12 mg/mL) and (P2) (4.20 mg/mL) so that the final concentration of template would make up 298 µM in the NMR tube. The solutions were allowed to equilibrate for 24 hours and the respective ^{31}P NMR spectra were recorded at RT.

Kinetics

The hydrolysis of nitrophenyl phosphate **1** (NPP) was monitored using UV-Vis spectroscopy: (λ_{max}= 405 nm, ε_{max}= 16963 $M^{-1}cm^{-1}$, in HEPES buffer (10 mM, pH 7.2). The chemical yield of the reaction was determined after a fixed time by converting the resulting absorbance into the true concentration values according to Beer-Lambert Law. The pH of each hydrolysis reaction was measured after 48 hours and 9 days.

Determination of the Amount of Product Formed in the Uncatalysed Reaction

A known amount of substrate was taken from the substrate stock solution and introduced into a glass test tube containing the reaction medium HEPES (10.0 mM, pH 7.2). The time at which the injection was made was taken as t = 0, and the absorbance of the sample recorded at 405 nm. The test tube was sealed with a rubber septum and placed in a water bath at 25 °C. The solutions were monitored at known time intervals and the absorbance values converted into concentration of the product **3** produced according to the Beer-Lambert Law. The yield was taken as a percentage, from the known amount of substrate in solution.

Determination of the Amount of Product Formed in the Presence of the Free Catalyst 4d and 21 used in excess

5 mM stock solutions of dichloride complexes **4a** and **23** were prepared in HEPES (10 mM, pH 7.2) in vials equipped with a magnetic stirring bar. Cobalt complexes were activated to the catalytically active aqua-hydroxo species **4d** and **21** by addition of 2 equivalents of 0.1 M NaOH. Activation of **4a** occurred rapidly as shown by the immediate change in the λ_{max} (ε) visible absorption band from 561 nm (134 $dm^3mol^{-1}cm^{-1}$) to 523 nm (127 $dm^3mol^{-1}cm^{-1}$), which was accompanied by colour change from violet to red, whereas activation of complex **23** required stirring at 50℃ for 1 hour followed by 1 hour at RT. This conversion from di-chloro (570 nm (224 $dm^3mol^{-1}cm^{-1}$) to aqua-hydroxo species (540 nm (214 $dm^3mol^{-1}cm^{-1}$) also accompanied by a significant colour change from violet to pink. The hydrolysis reaction was carried out under pseudo-first order conditions where the cobalt complex was used in 3 fold excess (1200 μM) over the NPP substrate **1** (400 μM). Each reaction sample was prepared by adding an aliquot from the Co(III) aqua-hydroxo complex stock solution into a cuvette containing HEPES buffer and completing the total volume (2.5 mL) with the addition of substrate at t = 0 min. Cuvettes were sealed and the samples were checked for absorbance at known time intervals and the obtained values were converted in yield of product **3** formed (%). The pH of the reaction solutions did not change significantly during the course of the hydrolysis reaction (pH 7.2±0.2).The final product % obtained was corrected for the background reaction. As expected the activity of **21** towards the hydrolysis of **1** was lower, with 67% of the substrate being hydrolysed after 48 hours compared to 75% for **4d** (Fig. S7)

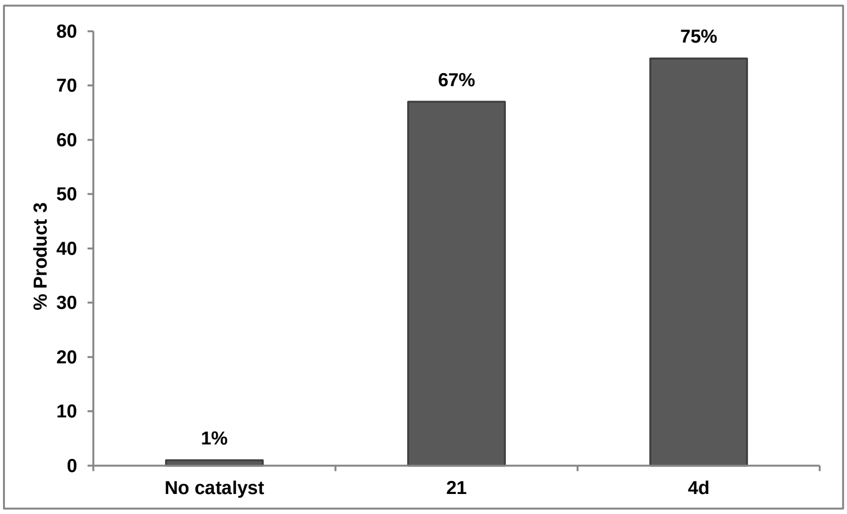

Fig. S8 Product **3** (%) formed after 48 hours (2 days) in the non-catalysed hydrolysis of phosphate **1** (400 μM), catalyzed by **21** (1200 μM) and by **4d** (1200 μM)

Kinetic Studies with Nanogels P1 and P2 and Catalysts 4d and 21

All the kinetic studies data shown in figure 4 were obtained using catalyst at 100 μM cobalt concentration.

a) Catalysts 4d and 21

In order to compare the amount of product produced after 9 days in the hydrolysis of substrate **1** catalysed by **4d** and **21** complexes, 5 mM stock solutions of **4a** and **23** were prepared in HEPES (10 mM, pH 7.2) and activated to form the active auqa-hydroxo species using 0.1 M NaOH as described earlier. The hydrolysis reaction was carried out under first order conditions where the cobalt complex was used in catalytic amounts (100 μM) over the NPP substrate **1** (442 μM). Each reaction sample was prepared by adding an aliquot from the Co(III) aqua-hydroxo complex stock solution into a test tube containing HEPES buffer and completing the total volume (3 mL) with the addition of substrate at t = 0 min. A solution containing only substrate **1** in HEPES was also prepared to determine the amount of product formed in the uncatalysed reaction after 9 days. The test tubes were sealed and the samples were checked for absorbance at known time intervals and the obtained values were converted into the amount of product formed (μM) using the Beer-Lambert Law. The pH of the reaction solutions did not change significantly during the course of the hydrolysis reaction (pH 7.2±0.2). The final amount of product (μM) obtained was corrected for the background reaction (Figure 4a).

a) Nanogels P1 and P2

The effect of the nanogels on the absorbance of the product was firstly tested by measuring the absorbance of a known concentration of *p*-nitrophenol **3** in 1.00 mg/mL solutions of the nanogels. No significant increase or decrease of the absorbance values was detected and therefore no corrections were necessary. For the catalytic tests, 2.00 mg/mL stock solutions of the polymers were used in HEPES (10.0 mM, pH 7.2). The polymers had previously been assessed by FAAS so that the cobalt content in each sample was known. Known amounts of **P1** and **P2** were measured into a separate test tubes containing HEPES buffer. The remaining volume was completed with the substrate to make up a 3 mL sample with a 100 µM concentration of cobalt. Solutions were prepared using substrate concentrations at 442 µM and at 664 µM. Upon addition of the substrate, the time was set to zero and the absorbance was measured. As had been done for the uncatalysed reaction, the absorbance values were monitored at given time intervals and solutions were kept in sealed test tubes in a water bath at 25 °C between measurements. The pH of the samples was always recorded after the measurement to allow the correction of the ε values and, consequently, the product concentration (figure 4b-d).

References

[1] J. P. Collman, P. W. Schneider, *Inorg. Chem.* **1966**, *5*, 1380-1384.
